# *Parp* mutations protect against mitochondrial dysfunction and neurodegeneration in a PARKIN model of Parkinson's disease

**DOI:** 10.1038/cddis.2016.72

**Published:** 2016-03-31

**Authors:** S Lehmann, A C Costa, I Celardo, S H Y Loh, L M Martins

**Affiliations:** 1 Cell Death Regulation Laboratory, MRC Toxicology Unit, Lancaster Road, Leicester LE1 9HN, UK

## Abstract

The co-enzyme nicotinamide adenine dinucleotide (NAD^+^) is an essential co-factor for cellular energy generation in mitochondria as well as for DNA repair mechanisms in the cell nucleus involving NAD^+^-consuming poly (ADP-ribose) polymerases (PARPs). Mitochondrial function is compromised in animal models of Parkinson's disease (PD) associated with PARKIN mutations. Here, we uncovered alterations in NAD^+^ salvage metabolism in *Drosophila parkin* mutants. We show that a dietary supplementation with the NAD^+^ precursor nicotinamide rescues mitochondrial function and is neuroprotective. Further, by mutating *Parp* in *parkin* mutants, we show that this increases levels of NAD^+^ and its salvage metabolites. This also rescues mitochondrial function and suppresses dopaminergic neurodegeneration. We conclude that strategies to enhance NAD^+^ levels by administration of dietary precursors or the inhibition of NAD^+^-dependent enzymes, such as PARP, that compete with mitochondria for NAD^+^ could be used to delay neuronal death associated with mitochondrial dysfunction.

PARKIN is an E3-ubiquitin ligase involved in mitochondrial quality control through the autophagic degradation of defective mitochondria (reviewed in ref. 1). Loss-of-function of PARKIN leads to mitochondrial dysfunction, which is a central pathogenic process in both familial and idiopathic forms of Parkinson's disease (PD) (reviewed in ref. 2). *Drosophila melanogaster* is a powerful model system by which to study the mechanisms of PD-associated neurodegeneration and to test therapeutic compounds *in vivo*. *Parkin* mutant flies show mitochondrial dysfunction associated with a loss of mitochondrial membrane potential (Δ*ψ*m).^3^ Other prominent features of *parkin* mutant flies include (1) the degeneration of the indirect flight muscle, resulting in a defective (crushed) thorax phenotype^4, 5^ and (2) the selective loss of dopaminergic neurons in the protocerebral posterior lateral 1 cluster.^6^

Nicotinamide adenine dinucleotide (NAD^+^) is involved in many key cellular processes and is important for both mitochondrial ATP production and the maintenance of redox levels via the control of NAD^+^/NADH ratios. Increasing NAD^+^ availability by either dietary supplementation of NAD^+^ precursors or inhibition of NAD^+^-consuming enzymes, such as poly (ADP-ribose) polymerase (PARP), has the therapeutic potential for different human disorders and age-associated diseases (reviewed in ref. 7, 8, 9). In models of mitochondrial dysfunction or metabolic impairment, increasing NAD^+^ availability through PARP inhibition or NAD^+^ precursors improves mitochondrial function and enhances oxidative metabolism and general fitness.^10, 11, 12, 13, 14^ This is reported to involve increased sirtuin activity that activates transcriptional regulators, such as PGC-1*α*, to increase mitochondrial content and metabolism, or to increase the mitochondrial unfolded protein response.^10, 11^ Sirtuins are also NAD^+^-consuming protein deacetylases with mono-ADP-ribosyltransferase activity (reviewed in ref. 15).

The effect of increasing NAD^+^ levels on mitochondrial function in PD is not well understood. Meanwhile, several studies using toxin-induced rodent models of PD have shown that inhibition of PARP rescues the degeneration of dopaminergic neurons in the substantia nigra pars compacta.^16, 17, 18^ PARPs are major NAD^+^-consuming nuclear proteins involved in repairing single-strand breaks in the DNA of healthy cells. During apoptosis, a form of programmed cell death, PARP is inactivated by proteolytic processing^19^ as a means to increase the bioavailability of NAD^+^^20^ and ATP for this energy-dependent form of cell suicide.

We report that in *Drosophila*, loss of *parkin* affects NAD^+^ metabolism. In addition, increasing NAD^+^ availability through dietary supplementation or by mutations in the *Parp* gene can improve mitochondrial function and PD-related phenotypes, as well as prevent neurodegeneration in *parkin* mutant flies.

## Results

### An NAD^+^-supplemented diet suppresses both mitochondrial dysfunction and neurodegeneration in *parkin* mutant flies

**Figure 1:**
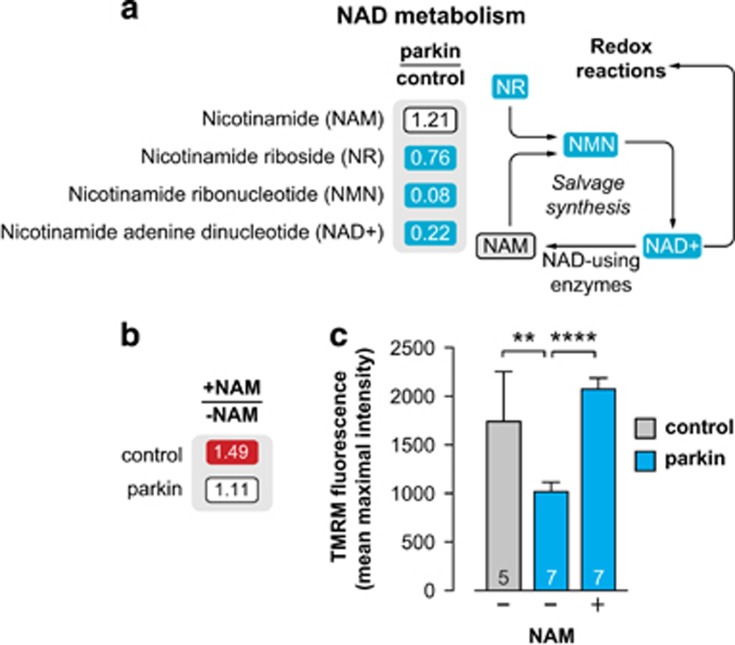
Dietary supplementation with NAM enhances mitochondrial function in *parkin* mutants. (**a**) Loss of *parkin* decreases NAD^+^ metabolites. Blue corresponds to metabolites that are significantly downregulated (*P*<0.05). The statistical significance was determined using Welch's two-sample *t*-test (*n*=8). (**b**) Dietary supplementation with NAM (5 mM) increases NAD^+^ levels in control wild-type flies. NAD^+^ levels were assessed using a colorimetric assay and normalised to total protein. The ratio of treatment on NAM to normal food is displayed. Red corresponds to a significant upregulation (*P*<0.05, two-tailed unpaired *t*-test; control *n*=6, parkin *n*=12). (**c**) Dietary supplementation with NAM (5 mM) reverses the loss of Δ*ψ*m in *parkin* mutants (mean±S.D.; asterisks, two-tailed unpaired *t*-test). Genotypes: control: *w*^*1118*^; parkin: *park*^*25*^/*park*^*25*^

To first explore if loss of Parkin affects NAD^+^ metabolism directly, we analysed the metabolic changes in *parkin* mutants. Cellular NAD^+^ pools are maintained through both *de novo* and salvage synthesis pathways (reviewed in ref. 21). The *de novo* pathway uses the amino acid tryptophan, as well as the NAD^+^ precursor vitamins nicotinic acid (also known as niacin or vitamin B3), its amide form nicotinamide (NAM), and nicotinamide riboside (NR), a lesser-known vitamin B3 available in selected foods, to generate NAD^+^. The salvage pathway recycles NAM, which is produced by NAD^+^-consuming enzymes such as PARP or sirtuins, to NAD^+^ via the intermediate metabolite nicotinamide mononucleotide (NMN). We detected decreases in both the NAD^+^ precursors NR and NMN as well as in total NAD^+^ levels in *parkin* mutant flies (Figure 1a).

NAD^+^ is an essential co-enzyme of the mitochondrial electron transport chain. As NAD^+^ levels were decreased in *parkin* mutants, we tested the effects of enhancing the NAD^+^ salvage pathway using NAM.

First we determined that a diet supplemented with NAM increased NAD^+^ levels in adult control flies (Figure 1b). When *parkin* mutants were exposed to a diet supplemented with NAM, NAD^+^ levels increase slightly but not significantly (Figure 1b); however, the loss of Δ*ψ*m (Figure 1c) was prevented indicating that an NAM-supplemented diet results in an improvement of mitochondrial function.

**Figure 2:**
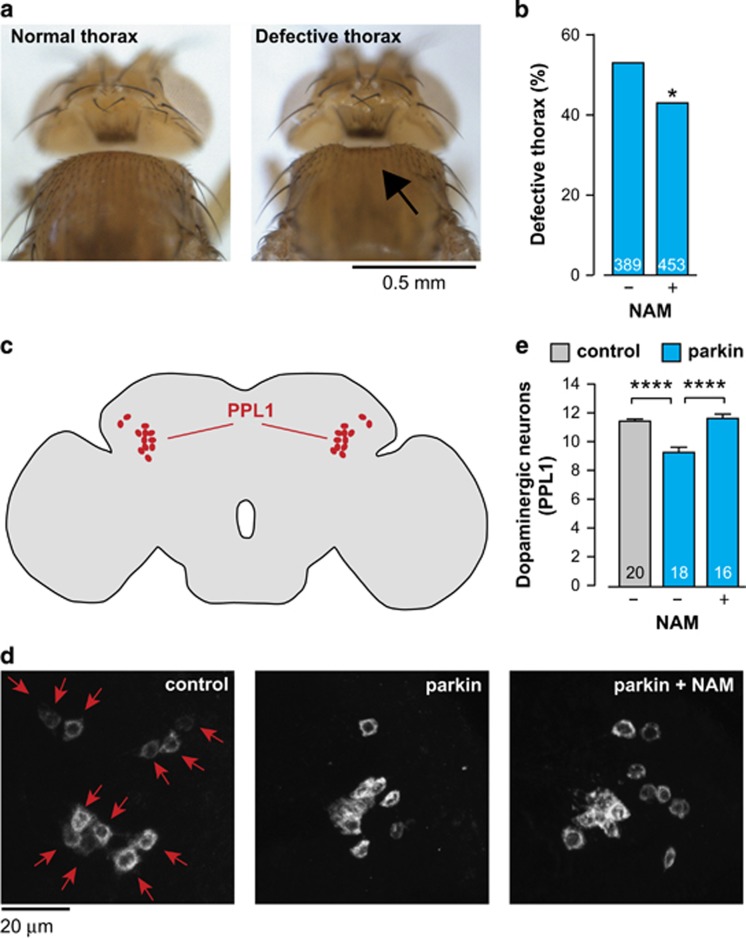
An NAM-enhanced diet blocks neurodegeneration in *parkin* mutants. (**a**) Representative images of normal and defective thorax in *parkin* mutants, the arrow points to a thoracic defect. (**b**) Dietary supplementation with NAM (5 mM) rescues the thoracic defects of *parkin* mutants (asterisks, *χ*^2^ two-tailed test, 95% confidence intervals). (**c**) Schematic diagram of a fly brain in sagittal orientation indicating the PPL1 cluster of dopaminergic neurons (red). (**d**) Anti-TH staining showing cell bodies of PPL1 neurons. Representative images for each condition are shown, arrows indicate individual PPL1 cluster neurons in a control animal. (**e**) Dietary supplementation with NAM (5 mM) rescues the loss of dopaminergic neurons in the PPL1 cluster of *parkin* mutant flies. (mean±S.E.M.; asterisks, two-tailed unpaired *t*-test). Genotypes: control: *w*^*1118*^; parkin: *park*^*25*^/*park*^*25*^

Maintaining *parkin* mutants on an NAM-supplemented diet reduced the appearance of the defective thorax phenotype (Figures 2a and b) and prevented the dopaminergic neuron loss (Figures 2c–e).

Collectively, these findings indicate that increasing the NAD^+^ pools through dietary supplementation with a form of vitamin B3 (NAM) improves mitochondrial function and is neuroprotective in *parkin* mutants.

### Mutation of *Parp* restores mitochondrial function in *parkin* loss-of-function mutants

**Figure 3:**
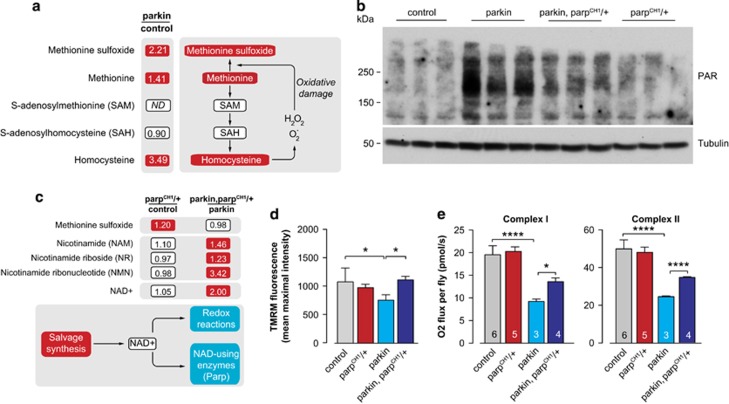
Mutation of *Parp* rescues mitochondrial function in *parkin* mutants. (**a**) Increased levels of oxidative stress-related metabolites in *parkin* flies. The metabolites indicated in red are significantly upregulated. ND corresponds to a metabolite below detection threshold. The statistical significance for fold-changes was determined using Welch's two-sample *t*-test (*n*=8). (**b**) Increased protein PARylation in *parkin* mutants is decreased upon a *Parp* mutation in *parkin, Parp*^*CH1*^*/+* double mutants. Whole-fly lysates were analysed using the indicated antibodies. Tubulin was used as a loading control. Three biological replicates are shown for each genotype. (**c**) Increased levels of NAD^+^ and NAD^+^ salvage metabolites and unchanged levels of oxidative stress marker methionine sulfoxide in *parkin*, *Parp*^*CH1*^*/+* double mutants. The metabolites indicated in red are significantly upregulated, as measured by metabolic profiling for the indicated comparisons of genotypes. Statistical significance was determined using Welch's two-sample *t*-test (*n*=8). (**d**) *Parp*^*CH1*^*/+* mutation prevents the loss of Δ*ψ*m in *parkin* mutants (mean±S.D.; asterisks, one-way ANOVA with Bonferroni's multiple comparison test, *n*=5). (**e**) *Parp*^*CH1*^*/+* mutation increases respiration in *parkin* mutants (mean±S.D.; asterisks, one-way ANOVA with Bonferroni's multiple comparison test). Genotypes: control: *w*^*1118*^; parkin: *park*^*25*^/*park*^*25*^; parkin, parp^CH1^/+: *park*^*25*^*, Parp*^*CH1*^/*park*^*25*^

Next, we investigated the aetiology of the decrease in NAD^+^ levels observed in *parkin* mutants. *parkin* mutant flies are reported to display enhanced oxidative stress,^6^ which is believed to activate PARPs to repair DNA damage, a process that leads to a depletion of cellular NAD^+^ stores.^22, 23, 24, 25^ Metabolic profiling of *parkin* flies confirmed the presence of enhanced levels of methionine sulfoxide, a marker of oxidative stress, as well as homocysteine, a metabolite that can generate reactive oxygen species upon auto-oxidation^26^ (Figure 3a). This was associated with an increase in protein PARylation (a post-translational protein modification carried out by PARPs that use NAD^+^ as a substrate) in *parkin* mutants (Figure 3b). We therefore questioned whether the observed depletion of NAD^+^ in *parkin* mutants could be caused by enhanced PARP activity. To address this, we examined whether a mutation of the *Parp* gene in *Parp*^*CH1*^*/+* flies^27, 28, 29^ could increase the NAD^+^ bioavailability in *parkin* mutants. We determined that a mutation of the *Parp* gene decreased PARylation in *parkin* mutants (Figure 3b), indicating that the *Parp* mutation decreases overall PARP activity. Next, we compared the metabolic profile of *parkin* with that of *parkin, Parp*^*CH1*^*/+* double mutants. We did not detect any alterations in NAD^+^ or its salvage precursors in *Parp*^*CH1*^/+ flies; however, *parkin*, *Parp*^*CH1*^*/+* double mutants showed increases in both NAD^+^ and its salvage metabolites when compared with *parkin* mutants alone (Figure 3c). Notably, protein levels of the oxidative stress marker methionine sulfoxide were unchanged in *parkin*, *Parp*^*CH1*^*/+* double mutants (Figure 3c).

We next examined the effects of *Parp* mutation on the mitochondrial phenotype of *parkin* mutants. When comparing *parkin* with *parkin*, *Parp*^*CH1*^*/+* double mutants, we observed that the *Parp* mutation led to a recovery of Δ*ψ*m (Figure 3d) and a restoration of respiratory activity of *parkin* mutants (Figure 3e).

### Mutation of *Parp* is neuroprotective in *parkin* mutant flies

**Figure 4:**
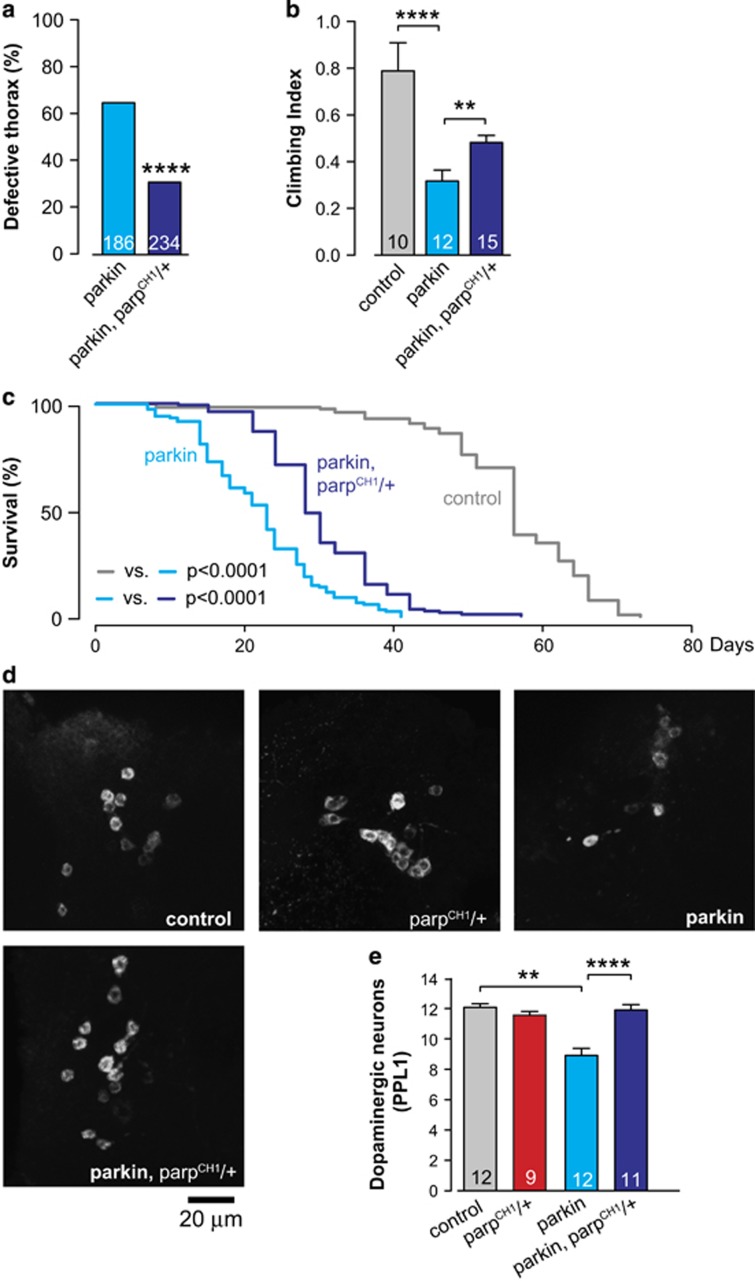
Mutation of *Parp* rescues *parkin* mutant phenotypes. (**a**) *Parp*^*CH1*^*/+* mutation rescues the thoracic defect (asterisks, *χ*^2^ two-tailed, 95% confidence intervals), (**b**) climbing ability (mean±S.D.; asterisks, two-tailed unpaired *t*-test), and (**c**) increases survival in *parkin* mutants (*n*=130 for control, *n*=122 for *parkin* and *n*=128 for *parkin*, *Parp*^*CH1*^*/+*; asterisks, log-rank, Mantel-Cox). (**d**) Anti-TH staining showing cell bodies of PPL1 neurons. Representative images for each genotype are shown. (**e**) *Parp*^*CH1*^*/+* mutation prevents the loss of dopaminergic neurons in the PPL1 cluster of *parkin* mutant flies (mean±S.E.M.; asterisks, one-way ANOVA with Bonferroni's multiple comparison test). Genotypes: control: *w*^*1118*^; parkin: *park*^*25*^/*park*^*25*^; parkin, parp^CH1^/+: *park*^*25*^*, Parp*^*CH1*^/*park*^*25*^

We next compared the phenotype of *parkin flies* with that of *parkin*, *Parp*^*CH1*^*/+* double mutants. This revealed that the *Parp* mutation is sufficient to reduce the defective thorax phenotype (Figure 4a), to improve the climbing performance (Figure 4b), and to enhance the survival (Figure 4c) in *parkin* mutants. In addition, although the mutation of *Parp* alone had no effect on dopaminergic neuron viability, the presence of a *Parp* mutation in *parkin* mutants was sufficient to rescue dopaminergic neuron loss (Figures 4d and e). Taken together, these data indicate that a mutation in the *Parp* gene in *parkin* mutants rescues mitochondrial dysfunction and is neuroprotective.

## Discussion

Impairment of mitochondrial function is one of the hallmarks of PD.^2^ Recent studies in fly models of PD have shown that vitamin K2, a mitochondrial electron carrier, and vitamin B12 (folic acid), a component of one-carbon metabolism, can rescue mitochondrial impairment and are neuroprotective.^3, 30^ Here, we further demonstrate the therapeutic potential of vitamin-based dietary interventions in an animal model of PD associated with mitochondrial dysfunction. Our data show that NAM, the amide form of vitamin B3 or niacin, suppresses neurodegeneration in *parkin* mutant flies. In support of this finding, an association between PD and niacin levels has been reported in two case–control studies, which found that a niacin-rich diet conferred a decreased risk of developing PD after correcting for occupational and environmental factors.^31, 32^ Furthermore, PD medication is associated with niacin deficiency, which may further aggravate PD pathogenesis.^33^ In addition, a patient study showed that low levels of NAD^+^/NADH and niacin were associated with decreased sleep quality and increased body pain in PD patients.^34^

A protective role for niacin was also demonstrated in several other animal models of PD. In *Drosophila*, NAM treatment improved climbing ability in *α*-synuclein transgenic flies,^35^ whereas in mice, NAM could rescue the MPTP-induced dopaminergic neuronal loss in the substantia nigra pars compacta.^36^ The present study extends these observations in a PARKIN model of PD by showing that the enhancement of NAD^+^ metabolism has a direct impact on mitochondrial function.

Oxidative damage has an important role in PD.^37^ This damage affects DNA molecules and activates PARP enzymes. The overactivation of PARPs, such as in a state of increased oxidative damage, is thought to deplete the stores of cellular NAD^+^ and thereby decrease its availability for other essential cellular processes. Conversely, the knockout of *Parp-1* in mice or its worm orthologue, *pme-1,* is reported to result in an increase in NAD^+^ levels.^10, 38^ This suggests that the total depletion of this NAD^+^-consuming enzyme improves the availability of this metabolite for other enzymatic reactions. Our fly data suggest that a mutation of *Parp* also increases NAD^+^ levels; although, this was only observed in *parkin* mutant flies and not in the heterozygous *Parp* mutants, possibly owing to an increased PARP activity in the *parkin* mutants. *Drosophila* that are homozygous for the *Parp*^*CH1*^ mutation die during the second instar larval stage;^28^ therefore, it is not possible to analyse NAD^+^ levels upon total loss of *Parp* in adult flies. Dietary supplementation with NAM failed to induce a significant increase in NAD^+^ in *parkin* mutants (Figure 1b) but rescued mitochondrial function (Figure 1c). We observed increased protein PARylation possibly owing to increased oxidative stress in these mutants (Figures 3a and b). Taken together these data indicate that Parp is overactivated in *parkin* mutants, therefore contributing to a depletion of NAD^+^ even when its salvage precursor NAM is added to their diet.

In summary, this work supports the concept that manipulating NAD^+^ levels by either dietary supplementation with biosynthetic precursors or through the inhibition of NAD^+^-consuming enzymes, such as PARPs, rescues mitochondrial function and is neuroprotective in models of mitochondrial dysfunction.

## Materials and Methods

### Genetics and *Drosophila* strains

Fly stocks and crosses were maintained on standard cornmeal agar media at 25 °C. The strains used were *park*^*25*^ (a kind gift from A Whitworth, MRC, Centre for Developmental and Biomedical Genetics, University of Sheffield, Sheffield, UK), *Parp*^*CH1*^ (a kind gift from V Corces, Department of Biology, Emory University, Atlanta, USA and A Tulin, Fox Chase Cancer Centre, Philadelphia, USA) and *w*^*1118*^ (Bloomington Stock Centre). *parkin, Parp*^*CH1*^/+ files were generated by recombining *park*^*25*^ with *Parp*^*CH1*^. All the experiments on adult flies were performed on males.

### Metabolic profiling

Global metabolic profiles were obtained from 3-day-old flies using the Metabolon Platform (Metabolon Inc., NC, USA) as previously described.^3^ Essentially, each sample consisted of eight biological replicates (100 flies per replicate). The sample preparation process was carried out using the automated MicroLab STAR system from Hamilton Company. For sample extraction, a 80% (v/v) methanol:water solution was used. Samples were then prepared for the appropriate instrument; either LC/MS or GC/MS. Compounds above the detection threshold were identified by comparison with library entries of purified standards or recurrent unknown entities. Identification of known chemical entities was based on comparison with metabolomic library entries of purified standards.

### Drug treatments

Nicotinamide (NAM) was incorporated into the fly food at a final concentration of 5 mM. Crosses were set up on normal food and transferred to NAM-containing food after 2 days. Larvae were treated with NAM throughout development. The adult flies were kept on drug-containing food throughout their lifespan, and they were transferred to vials with fresh food every 2–3 days.

### NAD^+^ assay

Total NAD^+^ levels were measured using an NAD^+^/NADH EnzyChrom colorimetric assay (BioAssay Systems, CA, USA). For NAD^+^ measurement, two 3-day-old flies were homogenised in the specified buffer on ice and processed according to manufacturer's instructions. NAD^+^ levels were normalised to total protein.

### Microscopy-based assessment of mitochondrial function

Measurements of Δ*ψ*m in fly brains were performed using tetramethylrhodamine (TMRM) in 3-day-old flies as previously described.^3^ In brief, fly brains were loaded for 40 min at room temperature with 40 nM TMRM in loading buffer (10 mM HEPES pH 7.35, 156 mM NaCl, 3 mM KCl, 2 mM MgSO_4_, 1.25 mM KH_2_PO_4_, 2 mM CaCl_2_, 10 mM glucose) and the dye was present during the experiment. In these experiments, TMRM is used in the redistribution mode to assess Δ*ψ*m, and therefore a reduction in TMRM fluorescence represents mitochondrial depolarisation. Confocal images were obtained using a Zeiss 510 confocal microscope equipped with a 40 × oil immersion objective. Illumination intensity was kept to a minimum (at 0.1–0.2% of laser output) to avoid phototoxicity and the pinhole was set to give an optical slice of 2 *μ*m. Fluorescence was quantified by exciting TMRM using the 565 nm laser and measured above 580 nm. Z-stacks of five fields of 300 *μ*m^2^ each per brain were acquired, and the mean maximal fluorescence intensity was measured for each group.

### Defective thorax analysis

Visual assessment of thoracic indentations (defective thorax) was assessed essentially as a binary assay: first we ask if a fly has a defective thorax or not; second we use *χ*^2^-statistics to determine whether the degree (percentage) of crushed thorax in the populations under analysis is significantly different.

### Analysis of dopaminergic neurons

Fly brains were dissected from 18-day-old flies and stained using anti-tyrosine hydroxylase (Immunostar, WI, USA) as previously described.^6^ Brain samples were placed in PBS+0.1% Triton in a coverslip clamp chamber (ALA Scientific Instruments Inc., NY, USA), positioned using a harp made of platinum wire and nylon string and imaged using confocal microscopy. Tyrosine hydroxylase-positive protocerebral posterior lateral 1 cluster neurons were counted per brain hemisphere. Data acquired for the assessment of each genotype were obtained as a single experimental set before statistical analysis.

### Protein extraction and western blotting

Protein extracts from whole flies were prepared by grinding flies in lysis buffer (100 mM KCl, 20 mM Hepes at pH 7.5, 5% (v/v) glycerol, 10 mM EDTA, 0.1% (v/v) Triton X-100, 10 mM DTT, 1 *μ*g/ml leupeptin, 1 *μ*g/ml antipain, 1 *μ*g/ml chymostatin and 1 *μ*g/ml pepstatin). The suspensions were cleared by centrifugation at 21 000 × *g* for 10 min at 4 °C, and protein concentrations of the supernatants were measured using the Bradford assay (Bio-Rad, CA, USA). All supernatants were mixed with 4 × LDS-loading buffer. For SDS-PAGE, equivalent amounts of proteins were resolved on 4–12% NuPAGE Precast Gels (Invitrogen, MA, USA) and transferred onto nitrocellulose membranes (Millipore, MA, USA) for *α*-PAR, or PVDF (Millipore) for *α*-Tubulin. The membranes were blocked in TBS (0.15 M NaCl and 10 mM Tris-HCl, pH 7.5) containing 5% (w/v) dried non-fat milk for 1 h at room temperature and probed with the indicated primary antibody before being incubated with the appropriate HRP-conjugated secondary antibody. Antibody complexes were visualised using Pierce's enhanced chemiluminescence system.

### Antibodies

Primary antibodies employed in this study were Tyrosine Hydroxylase (1 : 50, Immunostar), PAR (1 : 500, Trevigen, MD, USA. 4335-MC) and *α*-tubulin (1 : 5000, Sigma, Dorset, UK, T6074).

### Respirometry

Mitochondrial respiration in 3-day-old flies was assayed at 37 °C by high-resolution respirometry as previously described.^39^ OROBOROS Oxygraph DatLab software package (OROBOROS, Innsbruck, Austria) was used for the data acquisition (2 s-time intervals) and analysis, including the calculation of the time derivative of the oxygen concentration, signal deconvolution dependent on the response time of the oxygen sensor and correction for instrumental background oxygen flux. Respiration was assayed by homogenising two flies using a pestle in MiR05 respiration buffer (20 mM HEPES, 10 mM KH_2_PO_4_, 110 mM sucrose, 20 mM taurine, 60 mM K-lactobionate, 0.5 mM EGTA, 3 mM MgCl_2_, 1 g/l fatty acid-free BSA). Coupled state 3 respiration for complex I was assayed in MiR05 respiration buffer in the presence of 2 mM malate, 10 mM glutamate and 5 mM ADP. Complex II was assayed in respiration buffer supplemented with 1 mM rotenone, 10 mM succinate and 5 mM ADP.

### Climbing assay

The climbing assays were performed as previously described^4^ using a counter-current apparatus equipped with six chambers. A total of 15–20 male 3-day-old flies were placed into the first chamber, tapped to the bottom, and then given 20 s to climb a distance of 10 cm. The flies that successfully climbed 10 cm or beyond in 20 s were then shifted to a new chamber, and both sets of flies were given another opportunity to climb the 10-cm distance. This procedure was repeated a total of five times. After five trials, the number of flies in each chamber was counted. A video demonstrating the technique can be found at https://youtu.be/vmR6s_WAXgc. The climbing index was measured by using a weighted average approach using the following formula:


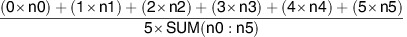


In this formula, n0 corresponds to the flies that fail the first trial and n1–n5 the number of flies that successfully pass each respective trial. At least 100 flies were used for each genotype tested.

### Lifespan analysis

Groups of 15 newly enclosed males of each genotype were placed into separate vials with food and maintained at 25 °C. The flies were transferred into vials containing fresh food every two to three days, and the number of dead flies was recorded. The data are presented as Kaplan–Meier survival distributions, and the significance was determined by log-rank tests.

### Statistical analyses

Descriptive and inferential statistical analyses were performed using GraphPad Prism 6 (www.graphpad.com). Computation of the minimal sample size for the variables measured in this study was assessed by power analysis, with an alpha initially set to 0.05, using StatMate 2 (www.graphpad.com).

Data are presented as the mean values, and the error bars indicate±S.D. or ±S.E.M. (as indicated). The number of biological replicates per experimental variable (*n*) is indicated in either the figure or the figure legends. Parametric tests were used (performed using data obtained from pilot experiments) after confirming that the variables under analysis displayed Gaussian distributions using the D'Agostino-Pearson test (computed using GraphPad Prism 6). The significance is indicated as **** for *P*<0.0001, *** for *P*<0.001, ** for *P*<0.01 and * for *P*<0.05. For the statistical analysis of metabolites in flies, pair-wise comparisons were performed using Welch's *t*-tests. The *q*-value provides an estimate of the false discovery rate according to Storey and Tibshirani.^40^ The investigators gathering quantitative data on biological samples were not blinded to the sample identities at the time of analysis. No specific randomisation strategies were employed when assigning biological replicates to treatment groups.

### Digital image processing

Western blot images were acquired as uncompressed, bitmapped digital data (TIFF format). Images were processed using Adobe Photoshop CS3 Extended, employing established scientific imaging workflows.^41^

## Abbreviations

NAD^+^: nicotinamide adenine dinucleotide
NAM: nicotinamide
PAR: poly (ADP-ribose)
PARP: poly (ADP-ribose) polymerase
PD: Parkinson's disease
PPL1: protocerebral posterior lateral 1
TMRM: Tetramethylrhodamine
Δ*ψ*m: mitochondrial membrane potential

## References

[bib1] Celardo I, Martins LM, Gandhi S. Unravelling mitochondrial pathways to Parkinson's disease. Br J Pharmacol 2014; 171: 1943–1957. 24117181 10.1111/bph.12433 PMC3976614

[bib2] Lehmann S, Martins LM. Insights into mitochondrial quality control pathways and Parkinson's disease. J Mol Med 2013; 91: 665–671. 23644494 10.1007/s00109-013-1044-y

[bib3] Tufi R, Gandhi S, de Castro IP, Lehmann S, Angelova PR, Dinsdale D et al. Enhancing nucleotide metabolism protects against mitochondrial dysfunction and neurodegeneration in a PINK1 model of Parkinson's disease. Nat Cell Biol 2014; 16: 157–166. 24441527 10.1038/ncb2901 PMC4199097

[bib4] Greene JC, Whitworth AJ, Kuo I, Andrews LA, Feany MB, Pallanck LJ. Mitochondrial pathology and apoptotic muscle degeneration in Drosophila parkin mutants. Proc Natl Acad Sci USA 2003; 100: 4078–4083. 12642658 10.1073/pnas.0737556100 PMC153051

[bib5] Park J, Lee SB, Lee S, Kim Y, Song S, Kim S et al. Mitochondrial dysfunction in Drosophila PINK1 mutants is complemented by parkin. Nature 2006; 441: 1157–1161. 16672980 10.1038/nature04788

[bib6] Whitworth AJ, Theodore DA, Greene JC, Benes H, Wes PD, Pallanck LJ. Increased glutathione S-transferase activity rescues dopaminergic neuron loss in a Drosophila model of Parkinson's disease. Proc Natl Acad Sci USA 2005; 102: 8024–8029. 15911761 10.1073/pnas.0501078102 PMC1142368

[bib7] Canto C, Menzies KJ, Auwerx J. NAD(+) Metabolism and the control of energy homeostasis: a balancing act between mitochondria and the nucleus. Cell Metab 2015; 22: 31–53. 26118927 10.1016/j.cmet.2015.05.023 PMC4487780

[bib8] Mouchiroud L, Houtkooper RH, Auwerx J. NAD(+) metabolism: a therapeutic target for age-related metabolic disease. Crit Rev Biochem Mol Biol 2013; 48: 397–408. 23742622 10.3109/10409238.2013.789479 PMC3858599

[bib9] Sauve AA. NAD+ and vitamin B3: from metabolism to therapies. J Pharmacol Exp Ther 2008; 324: 883–893. 18165311 10.1124/jpet.107.120758

[bib10] Bai P, Canto C, Oudart H, Brunyanszki A, Cen Y, Thomas C et al. PARP-1 inhibition increases mitochondrial metabolism through SIRT1 activation. Cell Metab 2011; 13: 461–468. 21459330 10.1016/j.cmet.2011.03.004 PMC3086520

[bib11] Canto C, Houtkooper RH, Pirinen E, Youn DY, Oosterveer MH, Cen Y et al. The NAD(+) precursor nicotinamide riboside enhances oxidative metabolism and protects against high-fat diet-induced obesity. Cell Metab 2012; 15: 838–847. 22682224 10.1016/j.cmet.2012.04.022 PMC3616313

[bib12] Cerutti R, Pirinen E, Lamperti C, Marchet S, Sauve AA, Li W et al. NAD(+)-dependent activation of Sirt1 corrects the phenotype in a mouse model of mitochondrial disease. Cell Metab 2014; 19: 1042–1049. 24814483 10.1016/j.cmet.2014.04.001 PMC4051987

[bib13] Felici R, Lapucci A, Cavone L, Pratesi S, Berlinguer-Palmini R, Chiarugi A. Pharmacological NAD-boosting strategies improve mitochondrial homeostasis in human complex I-mutant fibroblasts. Mol Pharmacol 2015; 87: 965–971. 25788480 10.1124/mol.114.097204

[bib14] Pirinen E, Canto C, Jo YS, Morato L, Zhang H, Menzies KJ et al. Pharmacological Inhibition of poly(ADP-ribose) polymerases improves fitness and mitochondrial function in skeletal muscle. Cell Metab 2014; 19: 1034–1041. 24814482 10.1016/j.cmet.2014.04.002 PMC4047186

[bib15] Houtkooper RH, Pirinen E, Auwerx J. Sirtuins as regulators of metabolism and healthspan. Nat Rev Mol Cell Biol 2012; 13: 225–238. 22395773 10.1038/nrm3293 PMC4872805

[bib16] Kim TW, Cho HM, Choi SY, Suguira Y, Hayasaka T, Setou M et al. (ADP-ribose) polymerase 1 and AMP-activated protein kinase mediate progressive dopaminergic neuronal degeneration in a mouse model of Parkinson's disease. Cell Death Dis 2013; 4: e919. 24232095 10.1038/cddis.2013.447 PMC3847323

[bib17] Wu XL, Wang P, Liu YH, Xue YX. Effects of poly (ADP-ribose) polymerase inhibitor 3-aminobenzamide on blood-brain barrier and dopaminergic neurons of rats with lipopolysaccharide-induced Parkinson's disease. J Mol Neurosci 2014; 53: 1–9. 10.1007/s12031-013-0175-5 24254220

[bib18] Yokoyama H, Kuroiwa H, Tsukada T, Uchida H, Kato H, Araki T. Poly(ADP-ribose)polymerase inhibitor can attenuate the neuronal death after 1-methyl-4-phenyl-1,2,3,6-tetrahydropyridine-induced neurotoxicity in mice. J Neurosci Res 2010; 88: 1522–1536. 19998477 10.1002/jnr.22310

[bib19] Lazebnik YA, Kaufmann SH, Desnoyers S, Poirier GG, Earnshaw WC. Cleavage of poly(ADP-ribose) polymerase by a proteinase with properties like ICE. Nature 1994; 371: 346–347. 8090205 10.1038/371346a0

[bib20] Kaufmann SH, Desnoyers S, Ottaviano Y, Davidson NE, Poirier GG. Specific proteolytic cleavage of poly(ADP-ribose) polymerase: an early marker of chemotherapy-induced apoptosis. Cancer Res 1993; 53: 3976–3985. 8358726

[bib21] Bogan KL, Brenner C. Nicotinic acid, nicotinamide, and nicotinamide riboside: a molecular evaluation of NAD+ precursor vitamins in human nutrition. Annu Rev Nutr 2008; 28: 115–130. 18429699 10.1146/annurev.nutr.28.061807.155443

[bib22] Alano CC, Garnier P, Ying W, Higashi Y, Kauppinen TM, Swanson RA. NAD+ depletion is necessary and sufficient for poly(ADP-ribose) polymerase-1-mediated neuronal death. J Neurosci 2010; 30: 2967–2978. 20181594 10.1523/JNEUROSCI.5552-09.2010 PMC2864043

[bib23] Alano CC, Ying W, Swanson RA. Poly(ADP-ribose) polymerase-1-mediated cell death in astrocytes requires NAD+ depletion and mitochondrial permeability transition. J Biol Chem 2004; 279: 18895–18902. 14960594 10.1074/jbc.M313329200

[bib24] Virag L, Szabo C. The therapeutic potential of poly(ADP-ribose) polymerase inhibitors. Pharmacol Rev 2002; 54: 375–429. 12223530 10.1124/pr.54.3.375

[bib25] Ying W. NAD+ and NADH in cellular functions and cell death. Front Biosci 2006; 11: 3129–3148. 16720381 10.2741/2038

[bib26] Finkelstein JD. The metabolism of homocysteine: pathways and regulation. Eur J Pediatr 1998; 157(Suppl 2): S40–S44. 9587024 10.1007/pl00014300

[bib27] Ong CT, Van Bortle K, Ramos E, Corces VG. Poly(ADP-ribosyl)ation regulates insulator function and intrachromosomal interactions in Drosophila. Cell 2013; 155: 148–159. 24055367 10.1016/j.cell.2013.08.052 PMC3816015

[bib28] Tulin A, Stewart D, Spradling AC. The Drosophila heterochromatic gene encoding poly(ADP-ribose) polymerase (PARP) is required to modulate chromatin structure during development. Genes Dev 2002; 16: 2108–2119. 12183365 10.1101/gad.1003902 PMC186441

[bib29] Zhang P, Spradling AC. Insertional mutagenesis of Drosophila heterochromatin with single P elements. Proc Natl Acad Sci USA 1994; 91: 3539–3543. 8170943 10.1073/pnas.91.9.3539 PMC43615

[bib30] Vos M, Esposito G, Edirisinghe JN, Vilain S, Haddad DM, Slabbaert JR et al. Vitamin K2 is a mitochondrial electron carrier that rescues pink1 deficiency. Science 2012; 336: 1306–1310. 22582012 10.1126/science.1218632

[bib31] Fall PA, Fredrikson M, Axelson O, Granerus AK. Nutritional and occupational factors influencing the risk of Parkinson's disease: a case-control study in southeastern Sweden. Mov Disord 1999; 14: 28–37. 9918341 10.1002/1531-8257(199901)14:1<28::aid-mds1007>3.0.co;2-o

[bib32] Hellenbrand W, Boeing H, Robra BP, Seidler A, Vieregge P, Nischan P et al. Diet and Parkinson's disease. II: A possible role for the past intake of specific nutrients. Results from a self-administered food-frequency questionnaire in a case-control study. Neurology 1996; 47: 644–650. 8797457 10.1212/wnl.47.3.644

[bib33] Bender DA, Earl CJ, Lees AJ. Niacin depletion in Parkinsonian patients treated with L-dopa, benserazide and carbidopa. Clin Sci (Lond) 1979; 56: 89–93. 477187 10.1042/cs0560089

[bib34] Wakade C, Chong R, Bradley E, Thomas B, Morgan J. Upregulation of GPR109A in Parkinson's disease. PLoS One 2014; 9: e109818. 25329911 10.1371/journal.pone.0109818 PMC4201464

[bib35] Jia H, Li X, Gao H, Feng Z, Li X, Zhao L et al. High doses of nicotinamide prevent oxidative mitochondrial dysfunction in a cellular model and improve motor deficit in a Drosophila model of Parkinson's disease. J Neurosci Res 2008; 86: 2083–2090. 18381761 10.1002/jnr.21650

[bib36] Anderson DW, Bradbury KA, Schneider JS. Broad neuroprotective profile of nicotinamide in different mouse models of MPTP-induced parkinsonism. Eur J Neurosci 2008; 28: 610–617. 18702732 10.1111/j.1460-9568.2008.06356.x

[bib37] Zuo L, Motherwell MS. The impact of reactive oxygen species and genetic mitochondrial mutations in Parkinson's disease. Gene 2013; 532: 18–23. 23954870 10.1016/j.gene.2013.07.085

[bib38] Mouchiroud L, Houtkooper RH, Moullan N, Katsyuba E, Ryu D, Canto C et al. The NAD(+)/sirtuin pathway modulates longevity through activation of mitochondrial UPR and FOXO signaling. Cell 2013; 154: 430–441. 23870130 10.1016/j.cell.2013.06.016 PMC3753670

[bib39] Costa AC, Loh SH, Martins LM. Drosophila Trap1 protects against mitochondrial dysfunction in a PINK1/parkin model of Parkinson's disease. Cell Death Dis 2013; 4: e467. 23328674 10.1038/cddis.2012.205 PMC3563993

[bib40] Storey JD, Tibshirani R. Statistical significance for genomewide studies. Proc Natl Acad Sci USA 2003; 100: 9440–9445. 12883005 10.1073/pnas.1530509100 PMC170937

[bib41] Wexler EJ Photoshop CS3 Extended for Biomedical Research [DVD-ROM and online course]. Lynda.com, Inc: Ventura, 2008.

